# Ability of the respiratory ECMO survival prediction (RESP) score to predict survival for patients with COVID-19 ARDS and non-COVID-19 ARDS: a single-center retrospective study

**DOI:** 10.1186/s40560-023-00686-z

**Published:** 2023-09-01

**Authors:** Elias H. Pratt, Samantha Morrison, Cynthia L. Green, Craig R. Rackley

**Affiliations:** 1 Division of Pulmonary, Allergy, and Critical Care Medicine, Duke University School of Medicine, Durham, NC USA; 2 Department of Biostatistics and Bioinformatics, Duke University School of Medicine, Durham, NC USA

**Keywords:** COVID-19, ARDS, Venovenous ECMO, ROC curve, In-hospital mortality

## Abstract

The respiratory ECMO survival prediction (RESP) score is used to predict survival for patients managed with extracorporeal membrane oxygenation (ECMO), but its performance in patients with Coronavirus Disease 2019 (COVID-19) acute respiratory distress syndrome (ARDS) is unclear. We evaluated the ability of the RESP score to predict survival for patients with both non-COVID 19 ARDS and COVID-19 ARDS managed with ECMO at our institution. Receiver operating characteristic area under the curve (AUC) analysis found the RESP score reasonably predicted survival in patients with non-COVID-19 ARDS (AUC 0.76, 95% CI 0.68–0.83), but not patients with COVID-19 ARDS (AUC 0.54, 95% CI 0.41–0.66).

**Supplementary Information:**

The online version contains supplementary material available at 10.1186/s40560-023-00686-z.

## Background

The respiratory ECMO survival prediction (RESP) score is a clinical decision support tool used to predict survival for patients with respiratory failure supported with VV-ECMO [1]. Current guidelines recommend using the RESP score to identify patients with ARDS most likely to benefit from VV-ECMO support [2]. However, the ability of the RESP score to predict outcomes in patients with ARDS caused by COVID-19 is less clear [3–5].

## Methods

We conducted a single center retrospective observational cohort study comparing the ability of the RESP score to predict survival for patients with COVID-19 ARDS and patients with ARDS from other causes. The Duke Health System Institutional Review Board approved the study with a waiver of informed consent (IRB Pro00090196) prior to data collection. All patients supported with VV-ECMO in the Duke University Hospital Medical Intensive Care Unit (MICU) between January 1, 2009, and December 31, 2021, were eligible for inclusion. Patients were excluded if they were supported with ECMO for indications other than ARDS, were placed on ECMO as a bridge to lung transplant or post-lung transplant, were supported with ECMO > 48 h prior to admission at our institution, or were < 18 years old at the time of cannulation. Data were collected by primary chart review and included baseline demographic and clinical data at the time of cannulation, variables for calculation of Sequential Organ Failure Assessment (SOFA) and RESP scores, etiology of ARDS (i.e., COVID-19 ARDS or non-COVID-19 ARDS), and survival to hospital discharge. The primary aim was to assess the ability of the RESP score to predict survival to discharge using the area under the receiver operating characteristic curve (ROC AUC) and assess the association between the RESP score and survival.

Demographic and clinical characteristics are presented using the median with 25th and 75th percentiles (Q1, Q3) or count (percentage). A ROC curve was constructed for both COVID-19 ARDS and non-COVID-19 ARDS patients. The ROC AUC and 95% confidence interval (CI) are reported, and DeLong’s method was used to test for differences between the AUCs. A logistic regression model including an interaction term between RESP score and COVID-19 status was fit to determine the association between RESP scores and survival to discharge. The odds ratio (OR) and 95% CI are presented for COVID-19 ARDS and non-COVID-19 ARDS patients, as well as the p-value for interaction. R version 4.2.0 and SAS version 9.4 (SAS Institute, Inc., Cary, NC) were used for all analyses, and a p-value < 0.05 was considered statistically significant.

## Results

**Fig. 1:**
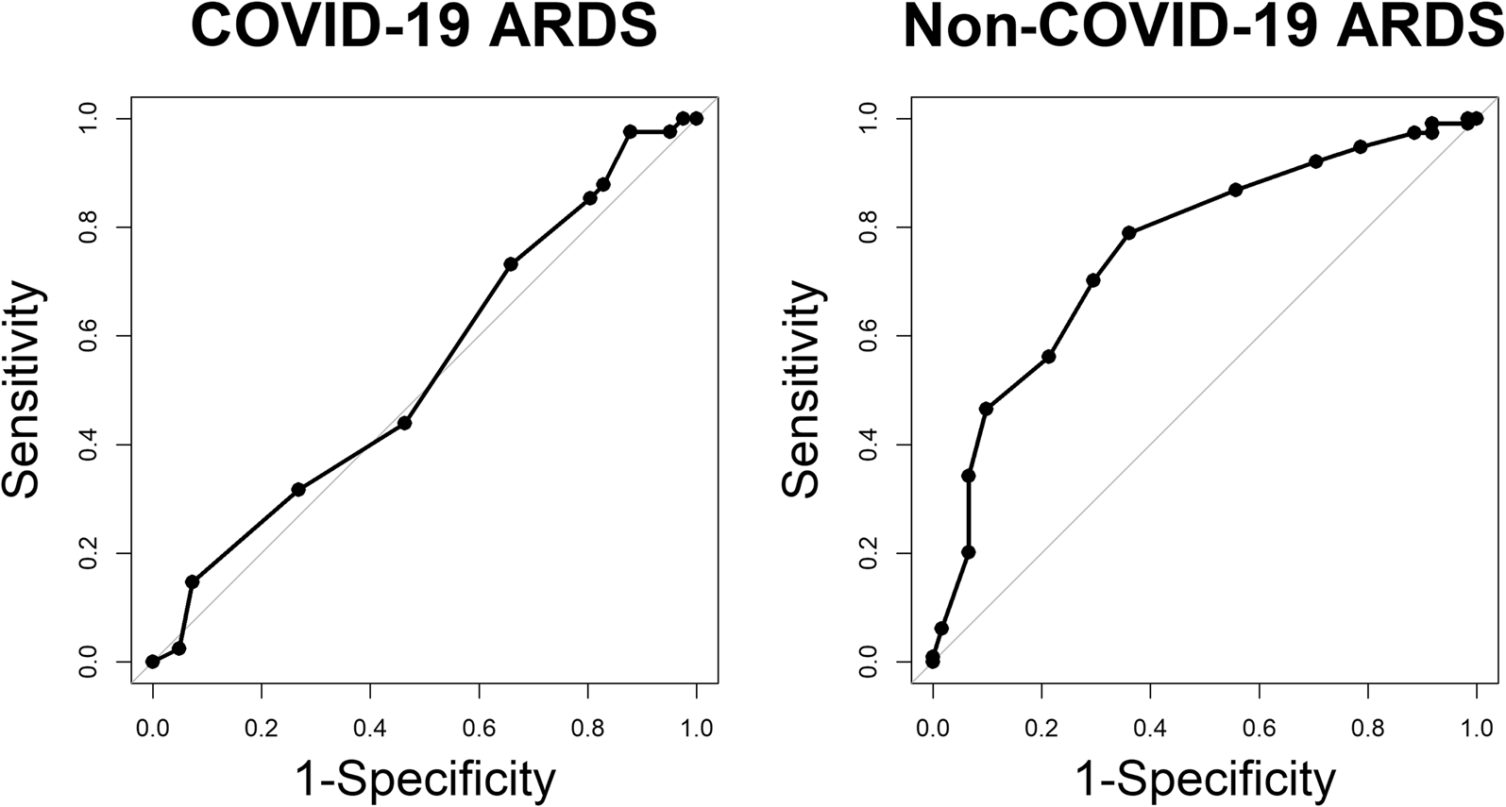
Respiratory ECMO survival prediction score receiver operating characteristic curves. RESP score ROC curves for patients with (**A**) COVID-19 ARDS and (**B**) non-COVID-19 ARDS. The AUC for patients with COVID-19 ARDS was 0.54 (95% CI 0.41–0.66) and for patients with non-COVID-19 ARDS was 0.76 (95% CI 0.68–0.83). DeLong’s test for difference between AUCs was significant (p-value = 0.003). *RESP*  respiratory ECMO survival prediction, *ROC*  receiver operating characteristic, *ARDS*  acute respiratory distress syndrome, *AUC*  area under the curve

There were 344 patients supported with VV-ECMO during the study period. Of these, 257 met inclusion criteria, including 175 with non-COVID-19 ARDS and 82 with COVID-19 ARDS (Additional file 1: Fig. S1). Baseline data for both cohorts are summarized in Table 1. The median (Q1, Q3) RESP score was similar between the non-COVID-19 ARDS and COVID-19 ARDS cohorts (3.0 [1.0, 5.0] vs 3.0 [2.0, 5.0]). The frequency of components composing the RESP score for each group are shown in Table 2. The ROC AUC for RESP score predicting survival to discharge was 0.54 (95% CI 0.41–0.66) for the COVID-19 ARDS cohort and 0.76 (95% CI 0.68–0.83) for the non-COVID-19 ARDS cohort, a statistically significant difference (p = 0.003) (Fig. 1). Higher RESP scores were significantly associated with survival to discharge in the non-COVID-19 ARDS cohort (OR 1.36, 95% CI 1.21–1.53, p < 0.001) but not in the COVID-19 ARDS cohort (OR 1.09, 95% CI 0.89–1.33, p = 0.39) (Additional file 1: Figs. S2, S3), though testing for an association between hospital survival and COVID-19 status was not significant (p-interaction = 0.065).

**Table 1:** Patient demographics and clinical characteristics

Characteristic	COVID-19 ARDS (N = 82)	Non-COVID-19 ARDS (N = 175)	Total (N = 257)	Missing, n (%)
Age (years)	43.6 (34.3, 50.4)	44.6 (33.0, 53.9)	44.3 (33.2, 52.7)	0 (0.0%)
Sex				0 (0.0%)
Female	32 (39.0%)	80 (45.7%)	112 (43.6%)	
Male	50 (61.0%)	95 (54.3%)	145 (56.4%)	
Race				0 (0.0%)
Black or African American	22 (26.8%)	51 (29.1%)	73 (28.4%)	
Multiple/other	5 (6.1%)	13 (7.4%)	18 (7.0%)	
White	38 (46.3%)	106 (60.6%)	144 (56.0%)	
Unknown/not reported	17 (20.7%)	5 (2.9%)	22 (8.6%)	
BMI (kg/m^2^)	34.9 (29.4, 40.9)	33.0 (27.3, 40.0)	33.5 (28.1, 40.4)	1 (0.4%)
PaO_2_/FIO_2_ ratio	75 (62, 88)	66 (53, 83)	69 (55, 85)	5 (1.9%)
Creatinine (mg/dL)	0.9 (0.7, 1.4)	1.6 (1.0, 2.8)	1.4 (0.8, 2.3)	1 (0.4%)
SOFA Score	7 (5, 9)	10 (8, 13)	9 (7, 12)	7 (2.7%)
RESP Score	3 (2, 5)	3 (1, 5)	3 (1, 5)	0 (0.0%)
Survival to discharge	41 (50.0%)	114 (65.1%)	155 (60.3%)	0 (0.0%)
Ventilator days prior to ECMO	5 (1, 8)	2 (1, 5)	3.0 (1.0, 6.0)	0 (0.0%)

Data are presented as median (Q1, Q3) or number (%)

*ARDS*  acute respiratory distress syndrome, *BMI*  body mass index, *FIO*_*2*_  fraction of inspired oxygen, *SOFA*  Sequential Organ Failure Assessment, *RESP*  Respiratory ECMO Survival Prediction

**Table 2:** RESP Score components by cohort

	COVID-19 ARDS (N = 82)	Non-COVID-19 ARDS (N = 175)	Total (N = 257)	Missing, n (%)
Age categories (years)				0 (0.0%)
18–49	60 (73.2%)	120 (68.6%)	180 (70.0%)	
50–59	18 (22.0%)	32 (18.3%)	50 (19.5%)	
≥ 60	4 (4.9%)	23 (13.1%)	27 (10.5%)	
RESP diagnoses				0 (0.0%)
Viral pneumonia	82 (100.0%)	70 (40.0%)	152 (59.1%)	
Bacterial pneumonia	0 (0.0%)	24 (13.7%)	24 (9.3%)	
Asthma	0 (0.0%)	1 (0.6%)	1 (0.4%)	
Trauma or burns	0 (0.0%)	6 (3.4%)	6 (2.3%)	
Aspiration pneumonitis	0 (0.0%)	38 (21.7%)	38 (14.8%)	
Other acute respiratory diagnosis	0 (0.0%)	36 (20.6%)	36 (14.0%)	
Non-respiratory or chronic respiratory diagnosis	0 (0.0%)	0 (0.0%)	0 (0.0%)	
Immunocompromised*	1 (1.2%)	19 (11.0%)	20 (7.8%)	2 (0.8%)
NMB prior to ECMO	80 (97.6%)	127 (72.6%)	207 (80.5%)	0 (0.0%)
Acute non-pulmonary infection	8 (9.8%)	21 (12.0%)	29 (11.3%)	0 (0.0%)
NO before ECMO	24 (30.0%)	35 (20.1%)	59 (23.2%)	3 (1.2%)
Bicarb infusion prior to ECMO	6 (7.3%)	35 (20.1%)	41 (16.0%)	1 (0.4%)
Cardiac arrest prior to ECMO	3 (3.7%)	22 (12.6%)	25 (9.7%)	0 (0.0%)
pCO_2_ ≥ 75 mm Hg	37 (45.1%)	47 (27.5%)	84 (33.2%)	4 (1.6%)
Peak pressure > 42 cm H_2_O	16 (19.5%)	24 (14.0%)	40 (15.7%)	3 (1.2%)
Ventilator prior to ECMO				0 (0.0%)
< 2 days	23 (28.0%)	85 (48.6%)	108 (42.0%)	
2–7 days	33 (40.2%)	64 (36.6%)	97 (37.7%)	
> 7 days	26 (31.7%)	26 (14.9%)	52 (20.2%)	

Data are presented as median (Q1, Q3) or number (%). *ARDS*  acute respiratory distress syndrome, *NMB*  neuromuscular blockade, *ECMO*  extracorporeal membrane oxygenation, *NO*  nitric oxide, *Bicarb*  bicarbonate

^*^Immunocompromised defined as any malignancy, solid organ transplant, HIV, or cirrhosis at the time of ECMO cannulation

## Discussion

Our results suggest the RESP score does not accurately predict in-hospital survival for patients with COVID-19 ARDS managed with VV-ECMO. In our COVID-19 ARDS cohort, the RESP score had a poor discriminative ability to predict survival and was not significantly associated with survival.

The reasons for the poor performance of the RESP score in our COVID-19 patients are unclear. Differences in the pathophysiology between COVID-19 ARDS and non-COVID-19 ARDS may reduce the clinical benefit of ECMO support for patients with COVID-19 ARDS. Alternatively, clinical variables not contained in the RESP score may better predict outcomes for patients with COVID-19 ARDS supported with ECMO. It is also possible that because the two study cohorts were treated exclusively in separate, consecutive time periods that differences in outcomes are related to changes in patient care (e.g. staffing shortages, increased patient volumes, different management practices) and not true differences between the cohorts.

Our study has several limitations. Its retrospective design makes it difficult to control for unmeasured confounding. Additionally, as our primary hypothesis was testing the discriminative ability of the previously published RESP score using a ROC curve, we did not adjust for other potential causes of poor outcomes in our models. As all patients were treated at a single center, its external validity may be limited.

In conclusion, the RESP score did not predict survival in patients with COVID-19 ARDS at our high volume ECMO center. Further studies are needed to confirm these findings in larger cohorts of patients with COVID-19 ARDS, especially those patients treated outside the height of the pandemic when shortages in medical staff and resources may have contributed to poor outcomes. Novel clinical decision support tools may be needed to identify patients with COVID-19 ARDS likely to benefit from VV-ECMO support in the future.

### Supplementary Information

**Additional file 1: Fig. S1.** CONSORT Diagram. **Fig. S2.** Box and whisker plots of RESP scores. **Fig. S3.** Association of RESP Score and survival to hospital discharge.

## Data Availability

The datasets used and/or analyzed during the current study are available from the corresponding author on reasonable request.

## Abbreviations

ARDS: Acute Respiratory Distress Syndrome
AUC: Area under the curve
BMI: Body Mass Index
CI: Confidence Interval
COVID-19: Coronavirus Disease 2019
ELSO: Extracorporeal Life Support Organization
FIO_2_: Fraction of Inspired Oxygen
MICU: Medical Intensive Care Unit
OR: Odds ratio
Q1: 25th Percentile
Q3: 75th Percentile
RESP: Respiratory ECMO Survival Prediction
ROC: Receiver Operating Characteristic
SOFA: Sequential Organ Failure Assessment
VV-ECMO: Venovenous Extracorporeal Membrane Oxygenation
