# Supplementary material for: Ability of the respiratory ECMO survival prediction (RESP) score to predict survival for patients with COVID-19 ARDS and non-COVID-19 ARDS: a single-center retrospective study
Source: J Intensive Care. 2023 Sep 1;11:37. doi: 10.1186/s40560-023-00686-z (PMC10472724; doi:10.1186/s40560-023-00686-z)
Supplement: Supplementary file 1 — Additional file 1: Fig. S1. CONSORT Diagram. Fig. S2. Box and whisker plots of RESP scores. Fig. S3. Association of RESP Score and survival to hospital discharge. [file 40560_2023_686_MOESM1_ESM.docx]

**Additional Digital Content**

**Table of Contents:**

[Figure S1. CONSORT Diagram. 2](#_Toc143608822)

[Figure S2. Box and whisker plots of RESP scores. 3](#_Toc143608823)

[Figure S3. Association of RESP Score and survival to hospital discharge. 4](#_Toc143608824)

# Figure S1. CONSORT Diagram.

*Some patients met multiple exclusion criteria

ECMO = extracorporeal membrane oxygenation, ARDS = acute respiratory distress syndrome, RESP = Respiratory ECMO Survival Prediction

**
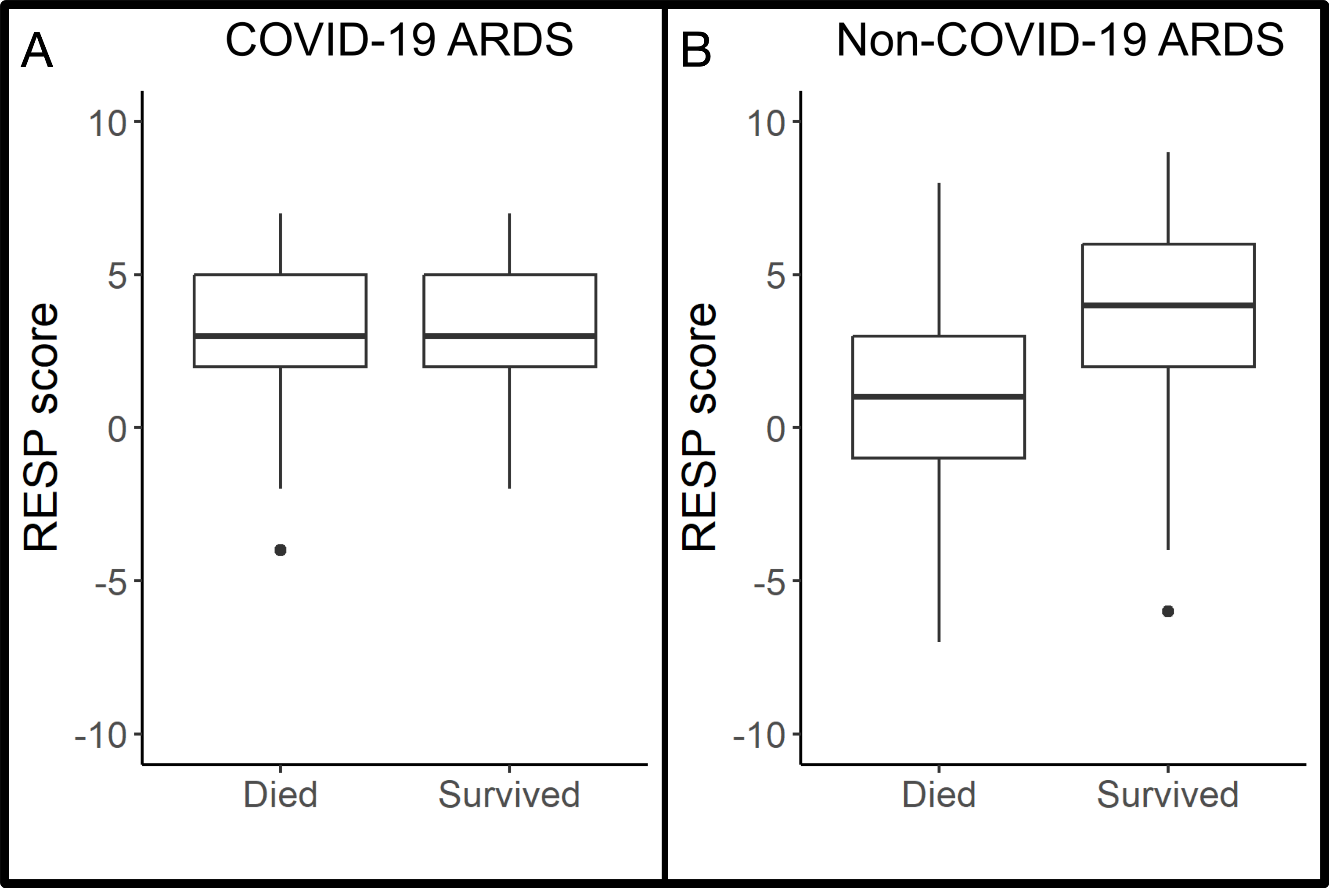
**

Figure S2. Box and whisker plots of RESP scores.

RESP scores grouped by survival to discharge in (a) non-COVID-19 ARDS and (b) COVID-19 ARDS. ARDS = acute respiratory distress syndrome, RESP = Respiratory ECMO Survival Prediction


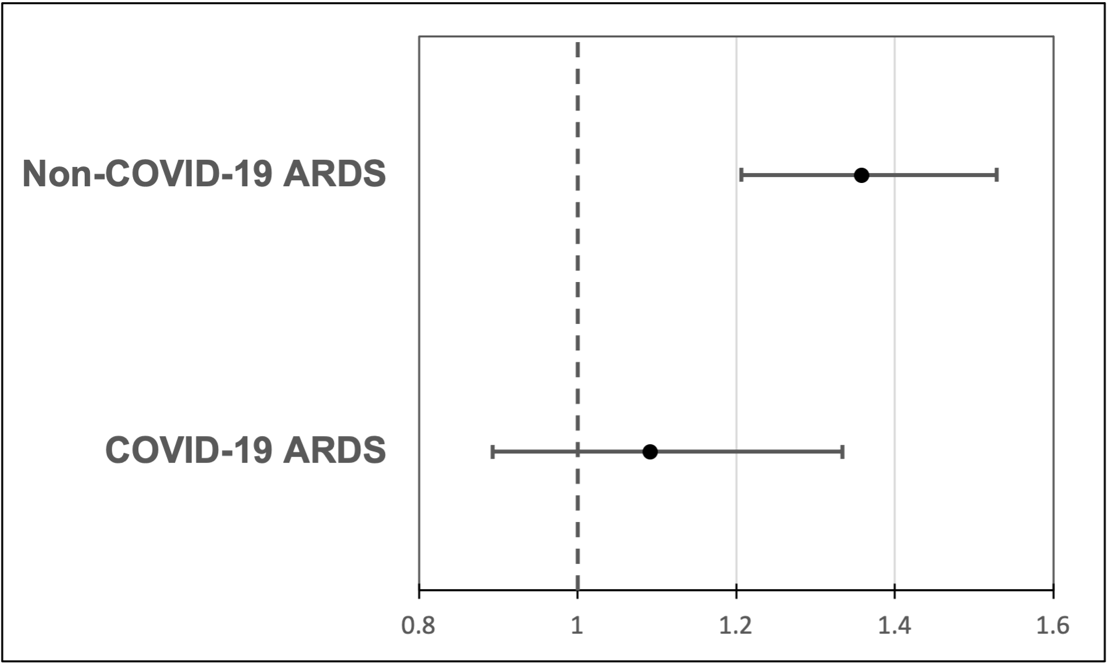


Figure S3. Association of RESP Score and survival to hospital discharge.

RESP score at time of cannulation was significantly associated with survival to discharge in the non-COVID-19 ARDS cohort (OR 1.36, 95% CI 1.21-1.53, p<0.001), but not in the COVID-19 ARDS cohort (OR 1.09, 95% CI 0.89-1.33, p=0.39). ARDS = acute respiratory distress syndrome, RESP = Respiratory ECMO Survival Prediction
